# Childhood interstitial lung diseases: current understanding of the classification and imaging findings

**DOI:** 10.1007/s11604-024-01603-6

**Published:** 2024-07-16

**Authors:** Yuko Tsujioka, Gen Nishimura, Eugene Nishi, Tatsuo Kono, Taiki Nozaki, Masahiro Hashimoto, Yoshitake Yamada, Masahiro Jinzaki

**Affiliations:** 1 Department of Radiology, Keio University School of Medicine, 35, Shinanomachi, Shinjuku-ku, Tokyo, 160-0016 Japan; 2 Department of Radiology, Musashino-Yowakai Hospital, Tokyo, Japan; 3 Department of Radiology, Keiyu Hospital, Yokohama, Japan; 4 Department of Radiology, Tokyo Metropolitan Children’s Medical Center, Tokyo, Japan

**Keywords:** Childhood interstitial lung diseases, chILDs, Pediatric interstitial lung disease, Diffuse lung disease in children, Surfactant disorder

## Abstract

Childhood interstitial lung diseases (chILDs) encompass a diverse group of disorders with a high mortality rate and severe respiratory morbidities. Recent investigations have revealed that the classification of adult ILDs is not valid for chILDs, particularly for ILDs of early onset. Therefore, Children’s Interstitial Lung Disease Research Cooperative of North America proposed a new classification of chILDs for affected children under 2 years of age, and later another classification for affected individuals between 2 and 18 years of age. In this review, we provide an overview of the imaging findings of chILDs by classification. Most infantile ILDs have unique clinical, radiological, and molecular findings, while the manifestation of pediatric ILDs overlaps with that of adult ILDs.

## Introduction

Childhood interstitial lung diseases (chILDs) encompass a diverse group of disorders diffusely affecting the pulmonary interstitial stroma and ultimately devastating the lung parenchyma. Although chILDs are individually rare, the prevalence may reach up to 16.2/100,000 [1–3]. However, most pediatricians and pediatric radiologists are not familiar with this category. Therefore, a substantial number of affected children remained undiagnosed. The clinical, radiological, histological, and genetic knowledge of chILDs has recently expanded, and it has become clear that the classification of adult ILDs is not valid for chILDs, particularly for congenital disorders in younger children. In 2007, the Children’s Interstitial Lung Disease Research Cooperative of North America proposed a new classification of chILDs for affected children under 2 years of age based on a retrospective review of 186 lung biopsies [4]. The classification was widely accepted and included in the 2013 American Thoracic Society Clinical Guidelines. In 2015, the working group of North America developed an extended classification for affected individuals between 2 and 18 years of age based on a retrospective review of 191 lung biopsies [5].

Both classifications are shown in Table 1. The former classification is comprised of the following major categories: (1) diffuse developmental disorders, (2) growth abnormalities, (3) surfactant dysfunction disorders and related abnormalities, and (4) specific conditions of unknown/poorly understood etiology, which include genetic diseases and developmental anomalies that overlap very little with adult ILDs. The latter is comprised of (1) disorders of the normal host, (2) disorders associated with systemic disease, (3) disorders of the immunocompromised host, and (4) disorders showing interstitial shadows associated with vascular abnormalities, in which many diseases overlap with adult ILDs, but the clinical manifestation may be unique in the pediatric age group.

**Table 1:** Classification of diffuse lung disease in childhood

Types of chILD	Comments
*More prevalent in infancy*	
**Diffuse developmental disorders**	
Acinar dysplasia	Arrest of alveolar maturation at the late 1st and early 2nd gestational ages
Congenital alveolar dysplasia	Arrest of alveolar maturation of at the late 2nd and early 3rd gestational ages
ACD-MPV	Malalignment of pulmonary veins with pre-acinar A-V shunt
**Alveolar growth abnormalities**	
Prenatal abnormalities	Pulmonary underdevelopment secondary to constriction of the thorax
Narrow thorax skeletal dysplasia, oligohydramnios, neuromuscular diseases, etc.	
Postnatal (chronic lung disease of prematurity)	Maldevelopment of premature lung due to O2 intoxication and barotrauma
Association with congenital cardiac disease	Maldevelopment of premature lung due to abnormal cardiopulmonary hemodynamics
Association with chromosomal abnormalities	Primary lung maldevelopment?
**Surfactant dysfunction and related abnormalities**	
SP-B gene deficiency	Impaired synthesis of SP-B causing DIP and PAP
SP-C gene deficiency	Impaired synthesis of SP-C causing NSIP, DIP, and PAP
ABCA3 deficiency	Impaired synthesis of lamellar bodies (surfactant complex) causing NSIP, DIP, and PAP
TTF-1 genetic mutations	Defective transcription of genes for surfactant synthesis and development of the brain/thyroid
Congenital GM-CSFα receptor deficiency	Congenital defect of GM-CSFαreceptor of alveolar macrophage causing PAP
Lysinuric protein intolerance	Cationic amino acids deficiency causing macrophage and immune dysfunction and PAP and IP
**Specific conditions of poorly defined etiology**	
NEHI	Pathognomonic imaging findings; transient hyperplasia of neuroendocrine cells
PIG	Mesenchymal immaturity, associated with other lung pathologies in infancy
**Miscellaneous monogenic chILDs**	
*Not specific to infancy and childhood*	
**Disorders of the normal host**	
BO associated with post infectious process	Viral infection-induced chronic small airway injury
Hypersensitivity pneumonitis	Alveolitis due to type3 or 4 hypersensitivity
Aspiration syndrome	
Eosinophilic pneumonitis	A heterogeneous group of disorders with eosinophilic lung infiltration
Idiopathic pulmonary hemosiderosis	Recurrent DAH of unknown etiology
**Disorders association with systemic disease**	
Immune related	
Goodpasture syndrome	DAH due to type 2 hypersensitivity induced by anti-GBM antibodies
Heiner syndrome	DAH due to cow’s milk allergy
Autoimmune pulmonary alveolar proteinosis	PAP due to autoantibodies against GM-CSFαreceptor of alveolar macrophage
Collagen vascular disease	
Non-immune-mediated systemic disorders	
Storage disease	Sphingolipid storage due to lysosomal enzyme deficiency
Sarcoidosis	Noncaseous granulomatosis
LCH	Proliferation of monoclonal dendric cells with increased MAPK/ERK signaling
Cystic fibrosis	Chronic airway injury due to increased mucus viscosity and recurrent infection
**Disorders of the immunocompromised host**	
Opportunistic infection	
Transplantation	Capillary leak/DAH at the acute stage and BO at the chronic stage
**Disorders masquerading as ILDs**	
Arterial hypertensive vasculopathy	Periarterial soft tissue proliferation?
Congestive vasculopathy	Obstruction of peripheral pulmonary veins
HHT	Multiple small AVFs superficially resembling ILD
Lymphatic disorders	
Lymphangiectasia	Impaired apoptosis of the embryonic lymphatic system
Lymphangiomatosis	Hamartomatous lymphatic proliferation
Pulmonary edema	

*ACD-MPV* alveolar capillary dysplasia with misalignment of pulmonary vein, *BO* bronchiolitis obliterans, *chILD* childhood interstitial lung disease, *DAH* diffuse alveolar hemorrhage, *DIP* desquamative interstitial pneumonia, *GM-CSFα* granulocyte macrophage colony stimulating factor alpha, *HHT* hereditary hemorrhagic telangiectasia, *ILD* interstitial lung disease, *IP* interstitial pneumonia, *LCH* Langerhans cell histiocytosis, *NEHI* neuroendocrine hyperplasia in infancy, *NSIP* non-specific interstitial pneumonia, *PAP* pulmonary alveolar proteinosis, *PIG* pulmonary interstitial glycogenosis, *TTF-1* thyroid transcription factor-1

The diagnosis of chILDs primarily rests on the histological findings of biopsy specimens. However, since advancement of molecular technology has elucidated the genetic basis of many chILDs, molecular diagnosis has become increasingly important. Nonetheless, it is likely that we only see the tip of the iceberg when it comes to the pathogenesis of chILDs [6].

Imaging examination plays a pivotal role in the clinical decision to diagnose children with chILDs. Although imaging alone is not enough to diagnose chILDs, the constellation of individually non-specific imaging findings can narrow the differential diagnosis. Imaging studies followed by genetic testing are the primary approach for chILDs of uncertain etiologies [6]. Needless to say, plain radiography is the first diagnostic tool. The technical development of MRI has enhanced its role in evaluating the lung morphology. However, CT is the gold standard in imaging for chILDs. We provide here an overview of the clinical and radiological characteristics of chILDs.

## Disorders in infancy

### Diffuse developmental disorders

This category includes acinar dysplasia (AcD), alveolar capillary dysplasia with misalignment of the pulmonary veins (ACD-MPV), and congenital alveolar dysplasia (CAD). These disorders present with severe respiratory distress and pulmonary hypertension shortly after birth. Affected neonates with AcD and ACD-MPV commonly succumb within hours or days after birth, while those with CAD only live for a few weeks and rarely into infancy [7, 8]. Lung transplantation is the only cure for affected children.

AcD and CAD result from premature cessation of lung maturation. Development of pulmonary alveoli is divided into the 5 stages, i.e., the embryonic stage (4–7 weeks), the pseudoglandular stage (5–17 weeks), the canalicular stage (16–26 weeks), the saccular stage (24 weeks to around birth), and the alveolarization stage (36 weeks to adulthood). The histological findings of AcD are identical to the immature alveoli ranging from the pseudoglandular to early canalicular stages. The histological characteristics are the same as that of “CPAM type 0” [7, 9]. In contrast, CAD has premature alveoli from the canalicular to early saccular stages [3]. It is tempting to assume that AcD and CAD are both on the spectrum of the same pathological condition, because they share the same genetic aberration, namely involvement of TBX4–FGF10–FGFR2 epithelial–mesenchymal signaling pathway [10, 11]. Imaging findings of AcD and CAD have been scarcely reported. As expected from their lung prematurity, they manifest with diffuse increased pulmonary opacities and reduced lung volume commonly complicated by air leaks, similar to those of “respiratory distress syndrome in newborns.”

**Fig. 1:**
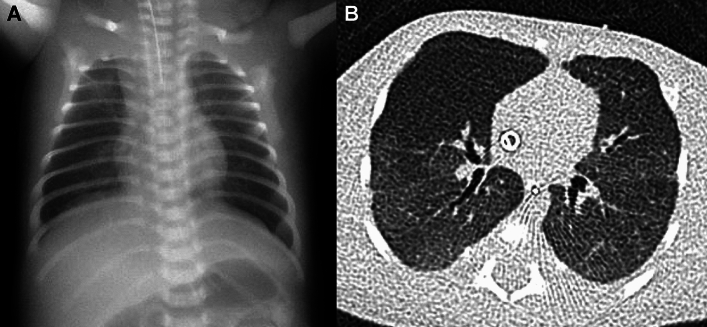
ACD-MPV. A neonate who presented with hypoxemia and hypercapnia immediately after birth, and later, severe pulmonary hypertension (less than 80% SpO_2_ despite NO inhalation). **A** CXR showed only mild hyperaeration and parahilar haziness in both lungs. **B** CT revealed increased lung densities that were mild but uniform

In contrast, ACD-MPV represents maldevelopment of pulmonary vasculatures. The disease name “misalignment of pulmonary veins” is a misnomer. The pathological hallmark is the presence of “misaligned and dilated bronchial veins” that bypass the peripheral pulmonary arteries of the bronchovascular bundles to the peripheral pulmonary veins of the interlobar septa. The anomalous veins are likely to be non-regressive remnants of embryonic broncho-pulmonary anastomotic circulation [12]. This pre-acinar shunt in pulmonary circulation is the pathogenesis of ACD-MPV that secondarily causes medial hypertrophy of pulmonary arteries, dysplasia of capillary vessels, and pulmonary lobular dysgenesis. Affected neonates usually develop hypoxemia at or shortly after birth, but rarely much later [3]. 40–90% of children with ACD-MPV have FOXF1 gene abnormalities and 10% are familial [3, 7, 13]. ACD-MPV is often complicated by other organ abnormalities, such as cardiac, gastrointestinal, and genitourinary anomalies [13, 14]. “Normal” development of alveoli in ACD-MPV yields normal CXR findings despite severe clinical symptoms (Fig. 1) [3].

### Alveolar growth abnormalities

This group of disorders are most common ILDs in neonates and in infants. The diverse diseases are either prenatal or postnatal in onset and commonly occur as a consequence of non-pulmonary diseases.

**Fig. 2:**
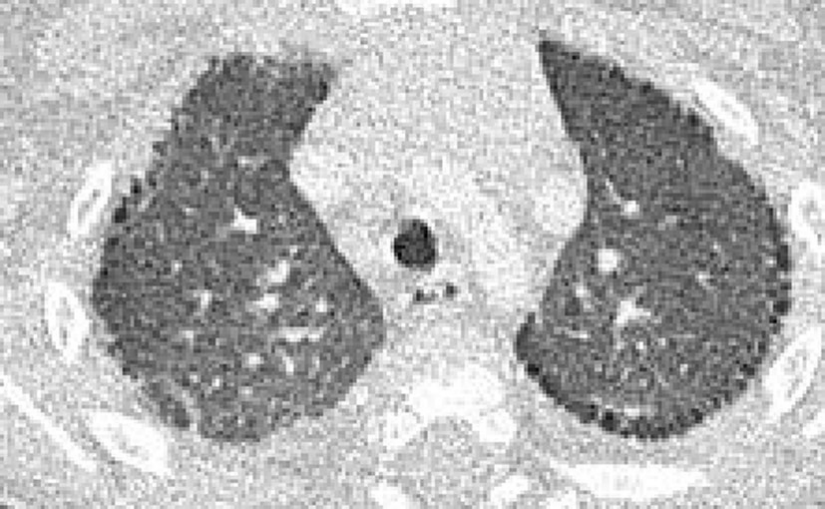
Alveolar growth abnormality due to trisomy 21. An infant with congenital heart disease. Preoperative CT unexpectedly uncovered small cysts seen extensively in the subpleural region and to a certain extent in the central zone of the lung

The prenatal alveolar growth abnormalities include pulmonary hypoplasia due to thoracic narrowing in skeletal dysplasias, thoracic restriction in oligohydramnios, reduced fetal thoracic movement in neuromuscular abnormalities, and lung compression in diaphragmatic hernia. Prenatal alveolar growth failure also occurs in congenital cardiac diseases and chromosomal abnormalities. It is speculated that the aberrant hemodynamics of pulmonary blood flow in utero interfere with alveolar development [14]. The pathogenesis of alveolar abnormalities in chromosomal aberration remains elusive; yet, it is known that the number of alveoli in trisomy 21 is reduced to 58–83% compared to that of normal children [15]. The histological characteristics of prenatal alveolar growth abnormalities are a marked decrease in the number of alveoli and enlargement of the alveolar ducts as a result of failure of alveolization of the alveolar and terminal bronchioles. The dilated alveolar ducts may be depicted as the characteristic finding of a cluster of small cysts prominent in the subpleural region on CT (Fig. 2) [16, 17].

**Fig. 3:**
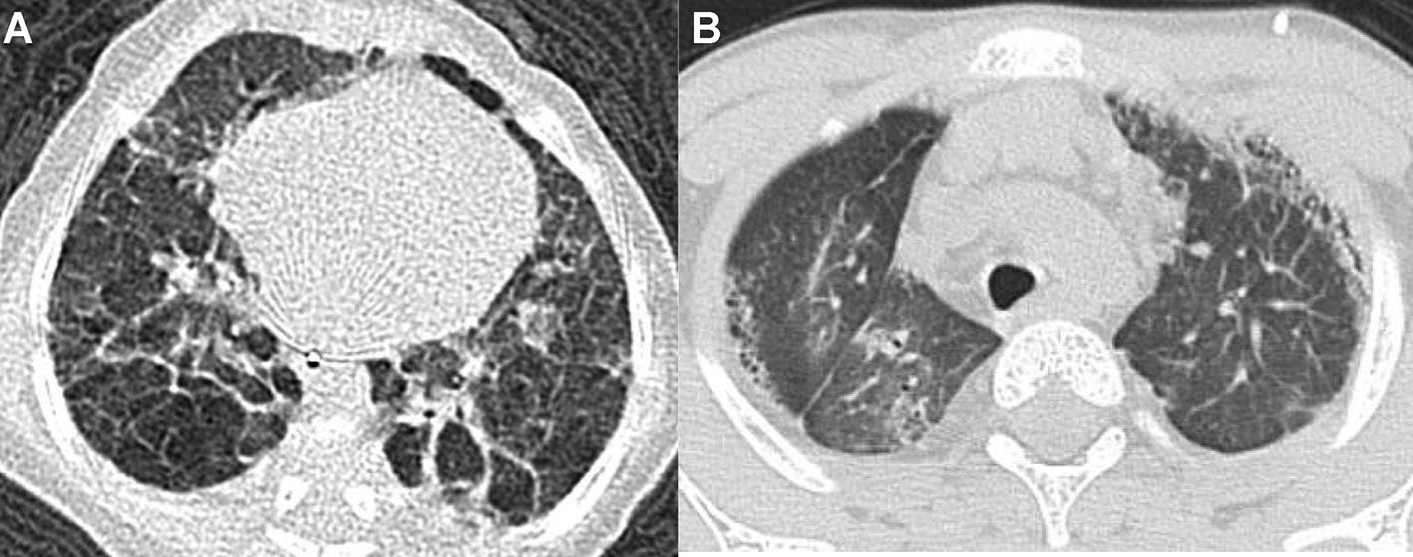
Alveolar growth abnormality due to broncho-pulmonary dysplasia (BPD) or chronic lung disease of prematurity (2 cases). **A** An infant prematurely born due to chorioamnionitis. CT at age 4 months showed inhomogeneous hyperlucency in both lungs associated with distorted bronchovascular shadows. **B** An 8-year-old boy with a history of chronic lung disease of prematurity. CT showed numerous subpleural microcysts that indicate a high likelihood of alveolar growth abnormality

The postnatal alveolar growth abnormalities are represented by broncho-pulmonary dysplasia (BPD) or chronic lung disease of prematurity [3, 4]. The histological changes of BPD primarily recapitulate those of the prenatal alveolar growth abnormalities [14]. However, BPD is complicated by various degrees of secondary airway narrowing and obstruction, resulting in hyperinflation, collapse, and fibrosis of the peripheral lungs. Radiologically, BPD is depicted as overinflated lungs with air cysts and subsegmental atelectasis. The radiological multiplicity is different from the imaging patterns in other types of alveolar growth abnormalities (Fig. 3).

### Surfactant dysfunction and related abnormalities

The alveolar surfactant consists of four proteins, surfactant protein (SP)-A, -B, -C, and -D, which are made in type II pneumocytes after 24 weeks of gestation and stored in lamellar bodies together with phospholipids. SP-B and SP-C, encoded by *SFTPB* and *SFTPC*, respectively, are highly hydrophobic proteins that bind to phospholipids and reduce the surface tension at the boundary between intraalveolar air and liquid along the alveolar surface, thereby decreasing a respiratory effort and preventing an alveolar collapse. SP-A and SP-D, on the other hand, are hydrophilic proteins mainly involved in the innate immune system and alveolar macrophage function [18]. ATP binding cassette transporter A3 (ABCA3) is involved in the intracellular transport of lipids to the lamellar bodies, and NKX2/TTF1 in the transcription of surfactant-related genes. Alveolar macrophages are pivotal to surfactant degradation.

SP-B gene deficiency is an autosomal recessive disorder with a poor prognosis, often progressing to death within a few months after birth. SP-B deficiency may secondarily interfere with normal SP-C production, resulting in a severe phenotype [19].

**Fig. 4:**
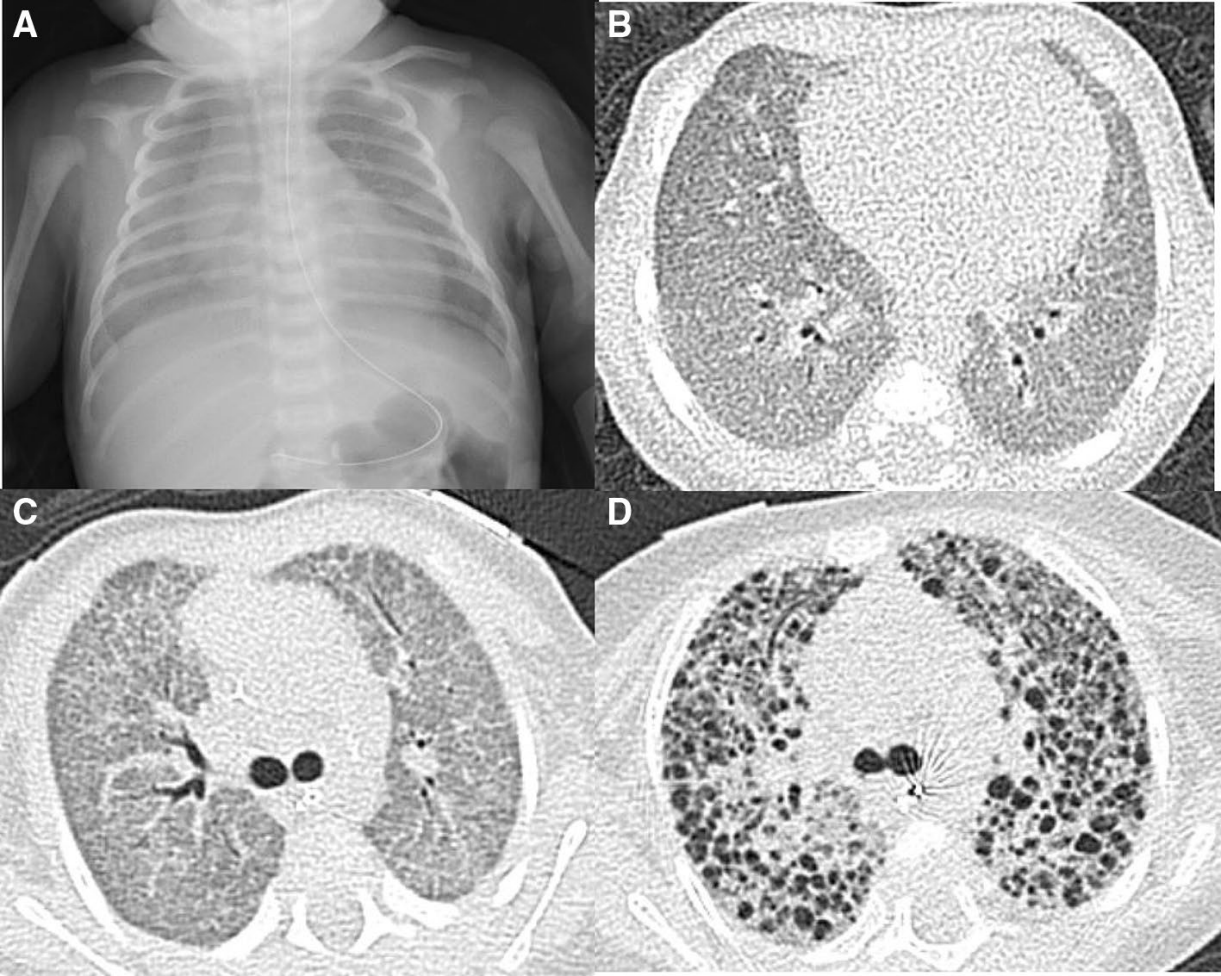
A severe manifestation of SP-C deficiency. A 1-month-old infant with failure to thrive and hypoxemia (70% of SpO_2_). **A**,**B** CXR and CT at age 1 month showed diffuse haziness and GGOs, respectively. The imaging findings look like those of respiratory distress syndrome in premature newborns. **C** CT at age 2 months showed thickening of interlobular septa and mild bronchial dilatation in addition to GGOs. **D** CT at age 4 months revealed development of multiple emphysematous cysts and progressive overinflation of the lung. The infant succumbed shortly after the CT examination

**Fig. 5:**
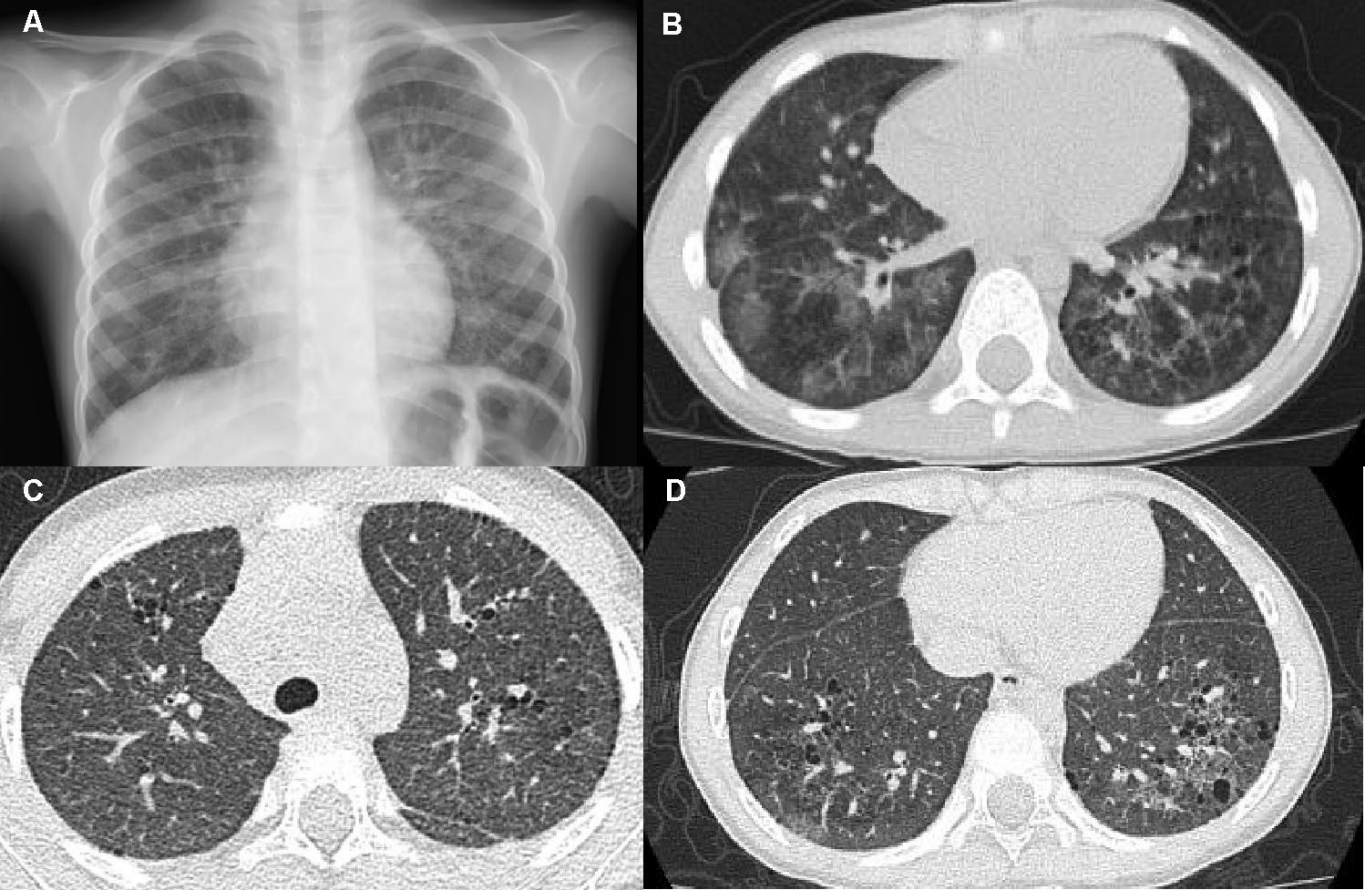
A mild manifestation of SP-C deficiency. A 5-year-old child with prolonged bloody sputum, triggered by viral respiratory infection. **A** CXR showed parahilar increased densities of both lungs. **B** CT displayed patchy GGOs intermingled with small cysts varying in size. **C**,**D** CT at age 8 years revealed clustering of much larger cystic lesions associated with mild GGOs in the central and peripheral zones of the lung

SP-C gene deficiency is inherited in an autosomal dominant manner and can occur as a result of de novo gene mutations. Symptoms may become apparent in neonates or infants, and airway infection may trigger the onset of the clinical symptoms. However, the clinical manifestations vary in severity. Interfamilial and even intrafamilial phenotypic variabilities are significant. SP-C deficiency may commence even in adulthood. The mild form represents a small subset of "unexplained" interstitial lung diseases in adulthood that manifest as an unusual pattern of interstitial pneumonia, i.e., exceptionally extensive cyst formation involving the entire lung on the background of a pattern of UIP and NSIP, singly or in combination (Figs. 4, 5) [20, 21].

ABCA3 deficiency is an autosomal recessive disorder that is the most frequent among the surfactant dysfunction and related disorders. The clinical course varies among affected children. Some children show relentless progression of respiratory failure and early demise, while others show spontaneous regression following transient respiratory morbidities. Based on the molecular data, frameshift and nonsense mutations have a poor prognosis, while missense mutations and intragenic insertion/deletion have a good prognosis [22].

*NKX2.1* encodes thyroid transcription factor-1 (TTF-1) that is expressed in the central nervous system, lung, and thyroid glands. TTF-1 deficiency is an autosomal dominant disorder with neurological dysfunction, respiratory distress, and primary hypothyroidism (brain-lung-thyroid syndrome). In the lung, TTF-1 is expressed in alveolar epithelium and is required for type II cell differentiation and expression of SP-B, SP-C, and ABCA3 [23]. The pulmonary manifestation varies among affected children, and early fatality may ensue. The presence of extrapulmonary phenotypes indicates a high likelihood of this disorder, and the molecular test can obviate the need for a lung biopsy to make the diagnosis.

The pathogenic basis of surfactant assembly disorders is complicated. The principle histological alterations are comprised of pulmonary alveolar proteinosis (PAP), desquamative interstitial pneumonia (DIP), and non-specific interstitial pneumonia (NSIP), singly or in combination. SP-B deficiency tends to present with DIP and PAP, while SP-C and ABCA deficiency tends to present with NSIP, DIP, and PAP. Histological findings include hyperplasia of type II alveolar epithelial cells, interstitial stromal thickening with a variable degree of inflammatory changes, intraalveolar accumulation of proteinaceous materials, and increased foamy macrophages with occasional cholesterol clefts [18, 24].

The imaging findings seen in these surfactant synthesis disorders reflect alveolar collapsibility and intraalveolar accumulation of surfactant materials. The typical clinical and radiological scenario is that “full-term neonates” show diffuse increased densities similar to those of respiratory distress syndrome seen in premature newborns. CT shows GGOs and interlobular septal thickening. Over time, the GGOs become less striking, and paraseptal emphysema-like small cysts can ensue [18]. The evolution from GGOs to multiple cysts does not necessarily indicate disease progression. The subpleural cysts are seen in asymptomatic children, and can develop without deterioration of respiratory function [6].

As opposed to surfactant synthesis disorders abnormal surfactant disassembly causes a pure type of PAP. Granulocyte macrophage colony stimulating factor alpha (GM-CSFα) is required for maturation and activation of alveolar macrophages that contribute to surfactant degradation [24]. Congenital deficiency of GM-CSFα receptor subunits 2A and B (encoded by *CSF2RA* and *CSF2RB*) on the surface of alveolar macrophages causes impaired surfactant degradation and accumulation of surfactant in the alveolar space, causing alveolar proteinosis. Its early childhood onset (pediatric IP) contrasts with the neonatal or infantile onset of surfactant synthesis disorders. The pathogenesis of GM-CSFα receptor deficiency resembles that of autoimmune PAP due to autoantibodies against GM-CSFα receptor. Autoimmune PAP is prevalent in adulthood, but rarely occurs in school-aged children. Both primary and secondary deficiency of GM-CSFα receptor are histologically identical to each other, and radiologically characterized by GGOs with smooth thickening of intralobular and interlobular septa, called a crazy paving appearance on CT.

**Fig. 6:**
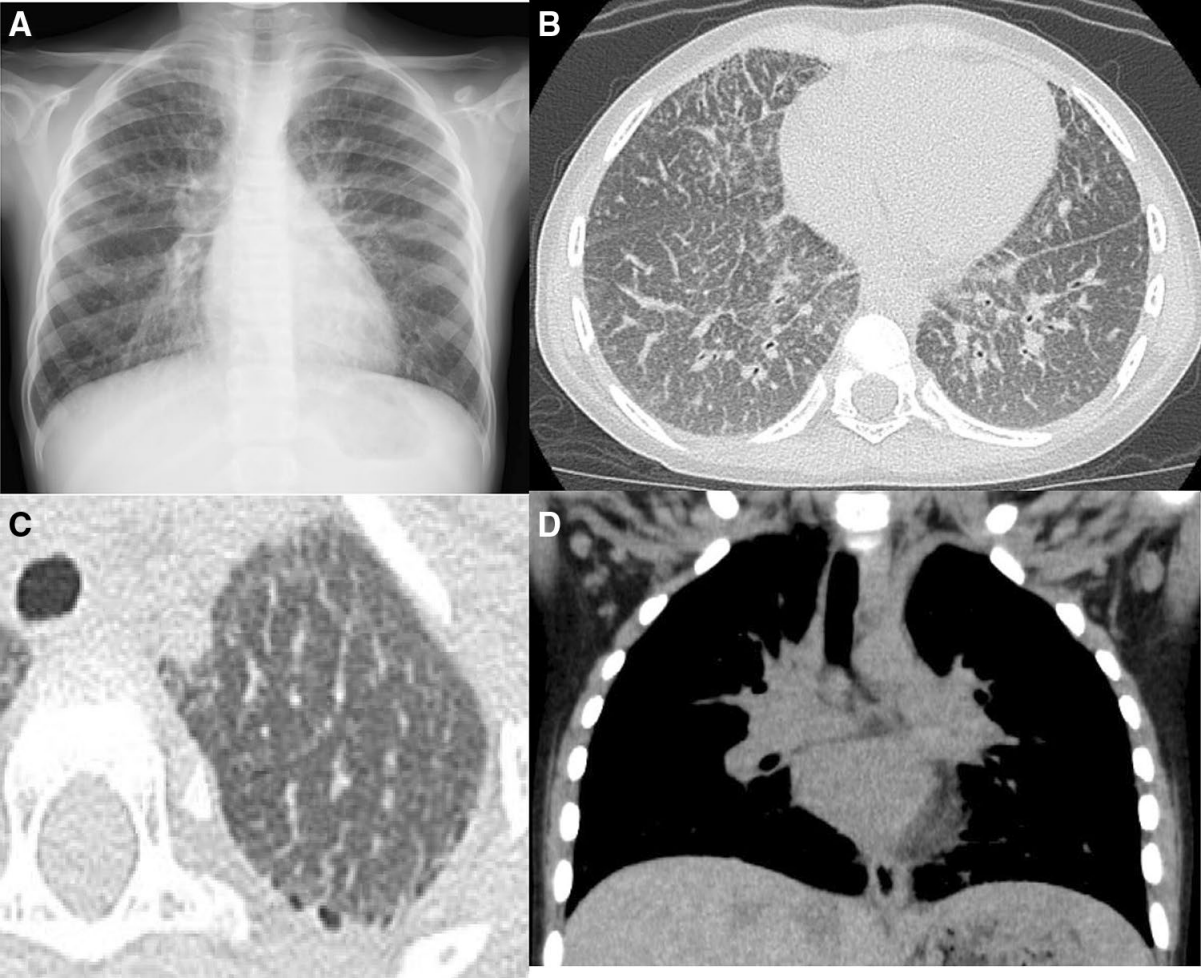
Lung involvement in lysinuric protein intolerance. An 8-year-old boy with postnatal growth failure, osteoporosis noted in early childhood, and aversion to protein rich food. The diagnosis was confirmed on molecular grounds. **A** CXR showed reticular shadows in both lungs. **B**,**C** CT revealed patchy GGOs and thickening of interlobular septa and bronchovascular bundles, reflecting alveolar proteinosis itself and fibrosis that developed over time. Presence of subpleural cysts suggests secondary alveolar growth impairment. **D** Mediastinal and hilar lymphadenopathy was evident

PAP is associated with lysinuric protein intolerance (LPI), a disorder characterized by impaired transport of cationic amino acids. LPI is an autosomal recessive disorder caused by mutations in *SLC7A7*. The abnormal transport of the amino acids interferes with the normal function of various organs. In the lung, it compromises alveolar macrophage activity and surfactant homeostasis. LPI is also complicated by immune dysregulation related to IL-1 and TNF. The dual involvement of surfactant disassembly and immune function causes PAP and chronic interstitial pneumonia. The disease process radiologically presents with diffuse GGOs, thickened septal walls, and subpleural and interlobar cysts. Honeycombing and enlarged lymph nodes are often observed (Fig. 6) [25, 26].

### Specific conditions of poorly defined etiology

**Fig. 7:**
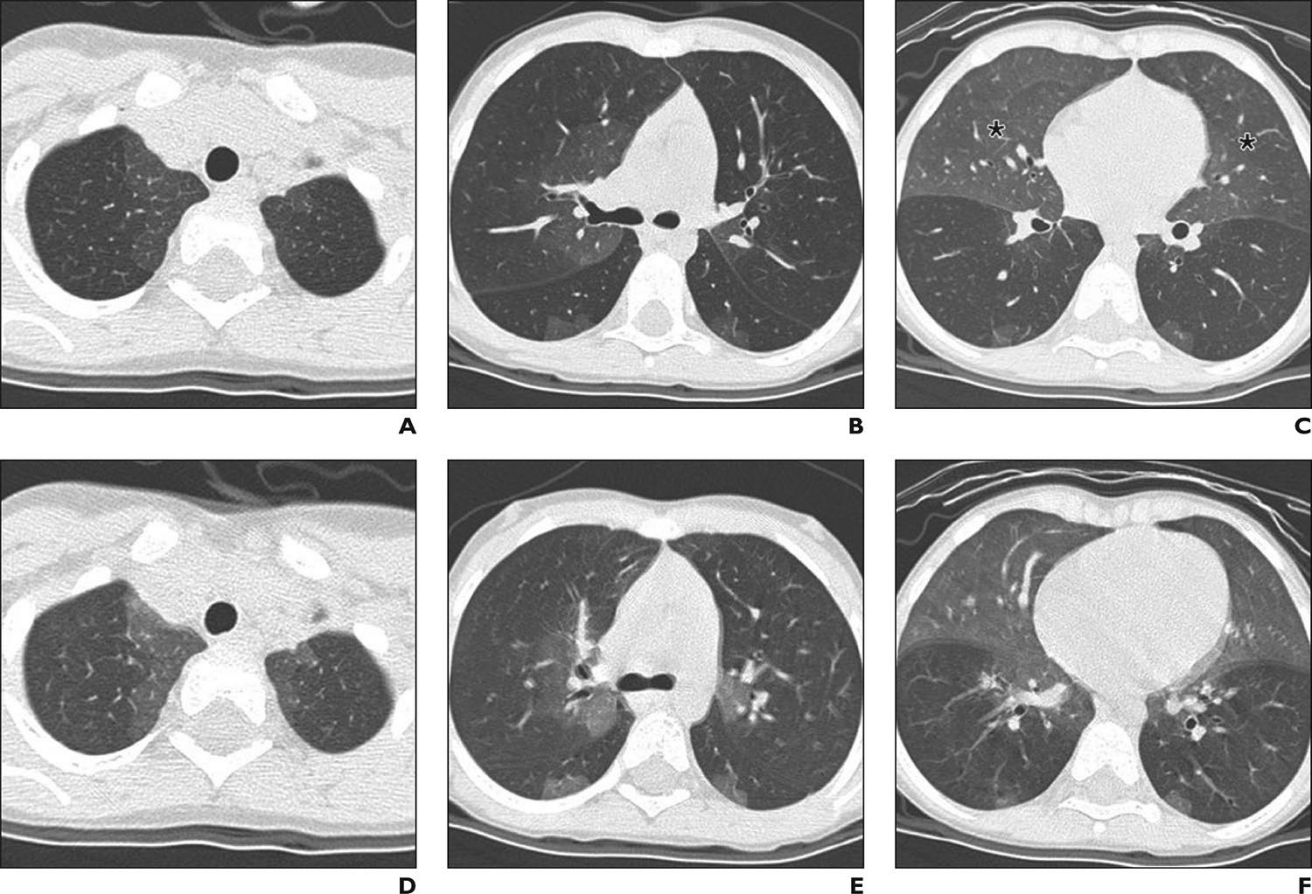
NEHI. A 40-month-old child. **A**–**C** Inspiratory CT showed relatively homogeneous GGOs without any structural distortion, particularly in the middle lobe, lingula, and paramediastinal regions. **D**–**F** Expiratory CT demonstrated only mild decrease in volume of both opaque and lucent areas, suggesting background air trapping of the entire lung (adapted from AJR 2010, Ref. [28])

Neuroendocrine hyperplasia in infancy (NEHI) was first proposed in 2005. The disorder had been previously referred to as “persistent tachypnea of infancy” [27]. Affected children present with prolonged tachypnea, retraction, crackles, and hypoxemia that usually start at less than 2 years of age, and most commonly in infancy (between 2 and 6 months of age). The respiratory insufficiency often requires oxygen administration; however, spontaneous resolution occurs in early childhood and the prognosis is generally favorable. The pathogenesis and genetic background are unresolved. Histological examination using HE staining does not reveal obvious morphologic abnormalities but mild hyperplasia of epithelial cells and smooth muscle cells. Immunostaining is crucial in revealing the characteristic hyperplasia of bombesin-positive airway endocrine cells [27, 28]. Nevertheless, the presence of neuroendocrine cells itself is a non-specific finding that can be seen in children with BPD, airway injury, lung hypoplasia, and even in normal children. In normal children, however, neuroendocrine cells exist only at a younger age and diminish over the years. Therefore, it is assumed that the pathogenesis in NEHI is a certain type of lung immaturity and delayed airway development [6]. Plain radiographs show only lung hyperinflation. However, CT demonstrates a characteristic finding, i.e., geographic GGOs mainly in the middle lobe and lingua without accompanying cystic changes (Fig. 7). The diagnostic sensitivity and specificity of CT are reported to be 78% and 100%, respectively. The pathognomonic CT findings can obviate the need for histologic examination and ineffective steroid and/or antimicrobial administration [29].

Pulmonary interstitial glycogenosis (PIG) was proposed in 2002 [30]. The condition is probably the same as what was previously referred to as cellular interstitial pneumonitis in infants (CIP), a respiratory disorder of unknown cause seen in infancy. PIG is histologically seen in association with a variety of disorders, such as alveolar growth abnormalities, BPD, pulmonary hypertension, and meconium aspiration syndrome. The histological characteristics of PIG include diffuse stromal thickening with increased lymphocytes and mesenchymal cells, but no fibrosis [31]. In recent years, PIG has not been considered a single disease entity, but a condition that reflects mesenchymal prematurity [6]. Prognosis depends on underlying comorbidities [28]. Imaging findings include normal or hyperinflated lung, widely spread GGO, interlobular septal thickening, reticular shadows, and sometimes cysts [32].

### Miscellaneous monogenic chILDs

**Fig. 8:**
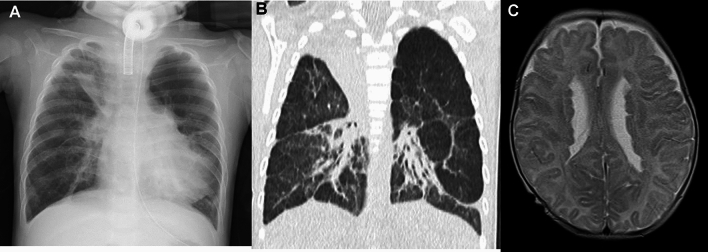
*FLNA*-associated ILD. A 5-month-old girl with biallelic *FLNA* mutations. **A**,**B** CXR and CT showed bilateral emphysematous changes associated with subsegmental atelectases predominating in the upper lungs. **C** T2-weighted brain MRI displayed periventricular nodular heterotopia

The *FLNA* gene encodes an actin-binding cytoskeletal protein termed filamin A. While the gain of function mutations of *FLNA* causes a few skeletal dysplasias, the loss of function mutations causes a diverse group of disorders, including periventricular nodular heterotopia, cardiovascular malformations, as well as interstitial lung disease. The lung disease is related to impaired alveolar growth, and is radiologically characterized by upper lobe overinflation, coarse pulmonary lobular septal thickening, and multiple patchy atelectasis (Fig. 8) [33, 34].

Other disorders include interstitial lung disease in STING-associated vasculopathy of infancy (SAVI) due to the gain of function of the *TMEM173* gene and *COPA*-related interstitial pneumonia, which belong to “interferonopathy” due to increased expression of interferon stimulated genes. JAK inhibitors may be beneficial to alleviate the clinical symptoms.

## Disorders not specific to infancy and childhood

### Disorders of normal host

Postinfectious and environment-induced conditions comprise this groups of disorders. Alveolar hemorrhage and pulmonary hemosiderosis belong to this group as well.

**Fig. 9:**
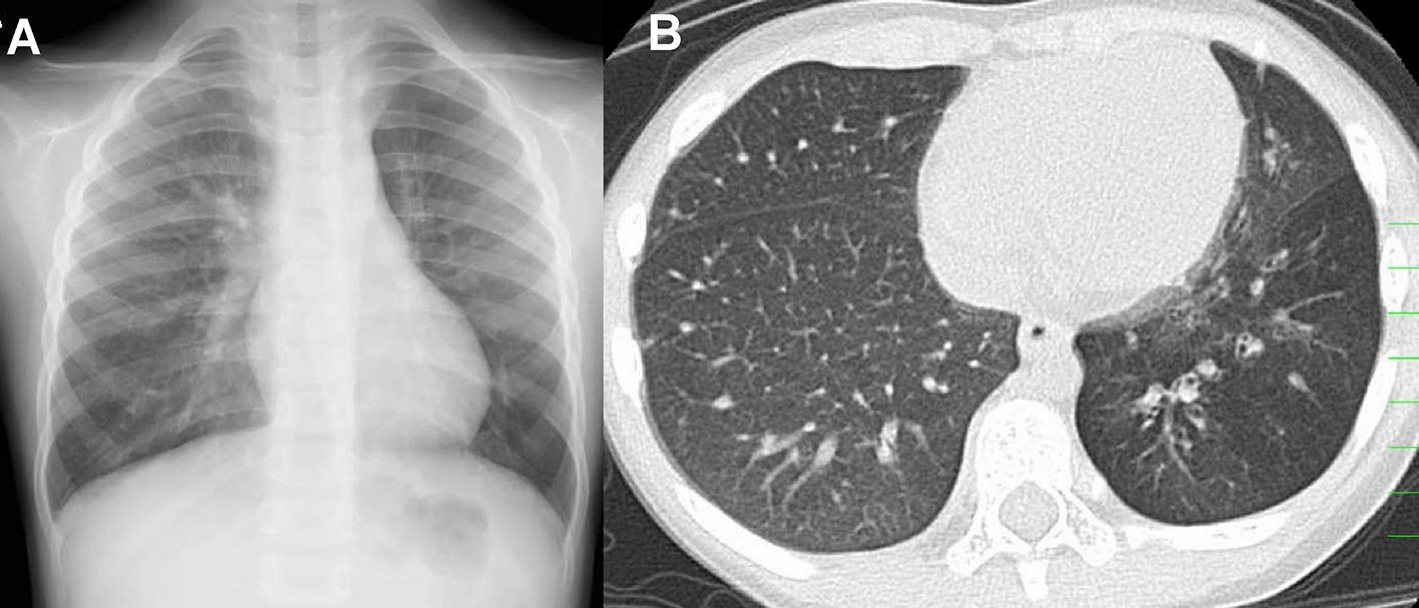
Swyer–James syndrome. A 4-year-old girl with a history of adenovirus pneumonia. **A** CXR showed unilateral lucent lung with reduced volume on the left side. **B** CT revealed generalized hyperlucency with attenuated vascular shadows and bronchial wall thickening of the left lung

Bronchiolitis obliterans (BO) is caused by postinfectious airway injury, and is the most common non-reversible pediatric small airway disease. Failure to eliminate the inflammatory process and persistent inflammatory cell infiltration following small airway infection ultimately cause damage, atrophy, and fibrosis of the bronchial epithelium [35]. Pathogens prone to BO include adeno, influenza, and RS viruses. Plain radiographs typically show unilateral, or asymmetrically bilateral, hyperlucent lung(s) with reduced volume in the affected lung. CT demonstrates characteristics of small airway injuries, such as attenuated pulmonary vascular shadows, mosaic attenuation, bronchial wall thickening, and bronchiectasis (Fig. 9). The mosaic attenuation reflects alveolar hyperinflation due to bronchial lumen narrowing and hypoxic vasoconstriction [36, 37]. BO can occur as a result of immune dysregulation after cardiopulmonary and bone marrow transplantations.

Hypersensitivity pneumonitis is allergic alveolitis caused by inhaled exogeneous agents. Immuno-complex (type III hypersensitivity) and cell-mediated (type IV hypersensitivity) responses are the primary pathogenesis of hypersensitivity pneumonitis in both children and adults. Hypersensitivity pneumonitis is divided into the acute and chronic forms. The acute form develops into fever, cough, and dyspnea within hours following exposure to high concentrations of caustic antigens. The chronic form is caused by prolonged exposure to low concentrations of antigens, and manifests in an insidious onset of exertional dyspnea and cough, weight loss, and fatigue. Imaging findings differ between the acute and chronic forms. Alveolitis of the acute form radiologically manifests as numerous centrilobular nodules and GGOs predominant in the middle and lower lung fields, while progressive fibrosis of the chronic form manifests as decreased lung volume, reticular shadows, GGOs, traction bronchiectasis, and cyst formation prominent in the middle lung field [38]. The prognosis of the chronic form is guarded. Although early diagnosis and elimination of the causal antigens are important, delayed diagnosis commonly occurs in pediatric hypersensitivity pneumonitis.

**Fig. 10:**
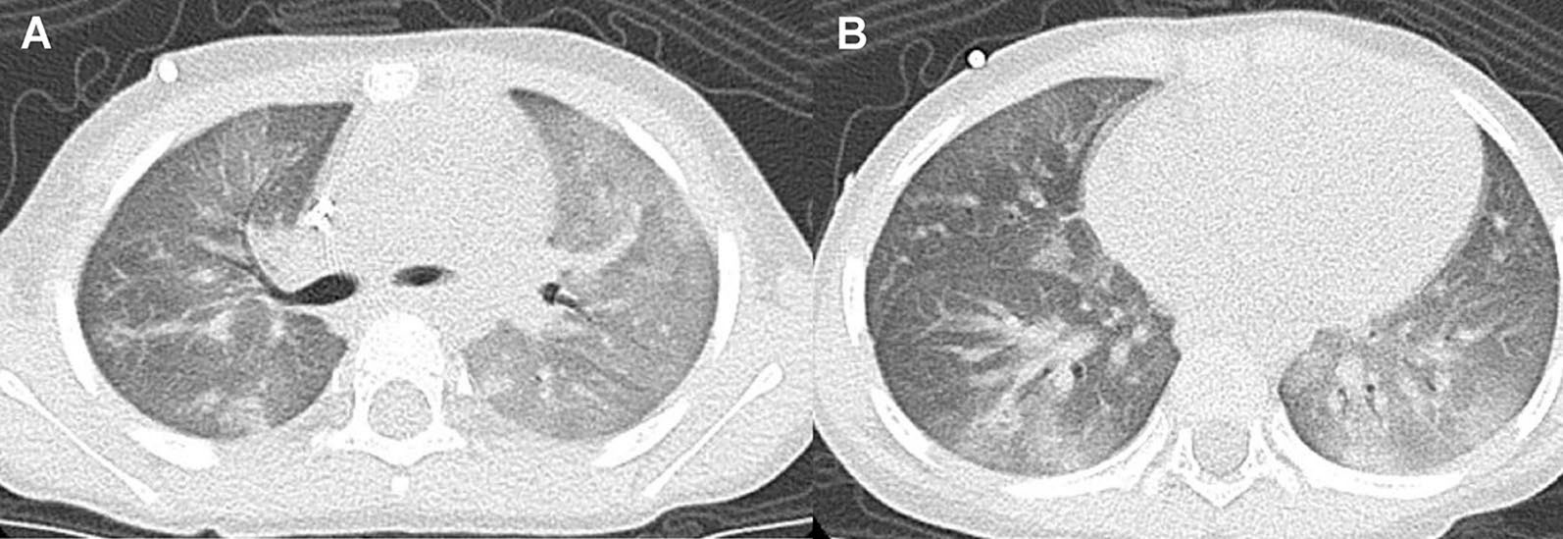
Post-transplant alveolar hemorrhage. A 3-year-old girl who developed hemoptysis after autologous peripheral blood stem cell transplantation following chemotherapy for neuroblastoma. **A**,**B** CT showed extensive ground glass shadows with “dark bronchus sign” (clearly depicted bronchial translucency due to alveolar blood filling)

Alveolar hemorrhage in children is divided into three categories: (1) idiopathic pulmonary hemosiderosis, (2) vasculitis/capillaritis, e.g., SLE, granulomatosis with polyangiitis, IgA vasculitis, and Heiner syndrome (milk allergy), and (3) miscellaneous causes, e.g., post-bone marrow transplantation, coagulation abnormalities, vascular lesions (AVM and Osler–Weber–Rendu disease), and cardiac diseases (mitral valve stenosis and pulmonary veno-occlusive disease (PVOD)) [39]. Idiopathic pulmonary hemosiderosis commonly becomes manifest in the first decade. The classic triad is bloody sputum, iron deficiency anemia, and opacity on CXR, which are usually not initially present. Persistent and/or repeated alveolar hemorrhage creates fibrosis of the lungs, pulmonary hypertension, and cor pulmonale. CT findings include GGOs, infiltrative shadows, and interlobular septal thickening (Fig. 10). The early manifestation may be a crazing paving appearance that later evolves into honeycomb lungs with traction bronchiectasis.

### Disorders related to systemic diseases

This diverse group of disorders are divided into immune-mediated and non-immune conditions. The former includes various types of vasculitis (e.g., Goodpasture syndrome, collagen-related vasculopathy, and Heiner syndrome) and rheumatologic disorders (collagen-vascular diseases), and autoimmune PAP due to autoantibodies to the GM-CSF receptor. The latter includes heterogeneous disorders, such as storage diseases, sarcoidosis, LCH, and cystic fibrosis.

Juvenile rheumatic diseases differ from adult collagen-vascular diseases in the clinical context. For example, juvenile systemic lupus erythematosus (JSLE) has a rapidly progressive clinical course and poorer prognosis than adult SLE.

**Fig. 11:**
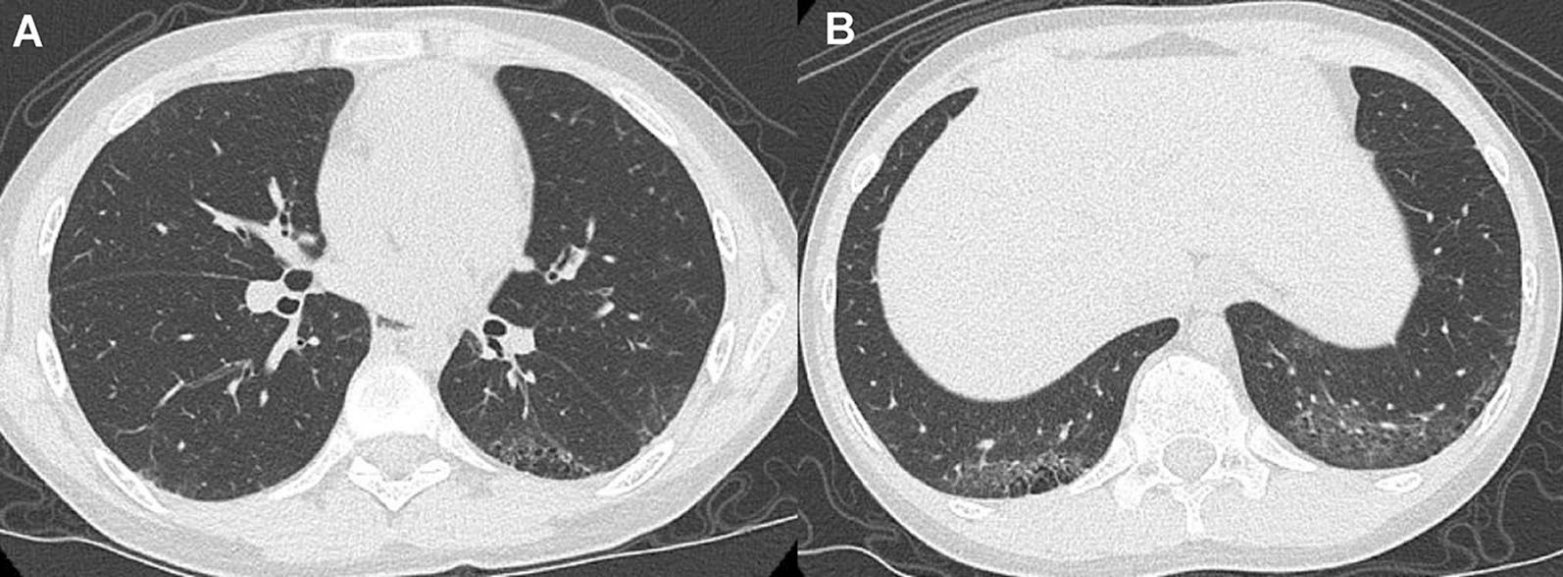
Pulmonary manifestation of SLE. A 17-year-old woman. **A**,**B** CT showed subpleural GGOs with cystic changes dependently distributed. Although the lung changes were relatively mild, the main pulmonary arteries were dilated due to lupus-associated pulmonary hypertension (not shown)

JSLE commonly manifests in late childhood and adolescence (the average age of onset: 11 years) and is much more common in girls. JSLE is more frequently complicated by lung and pleural involvement than adult SLE, which is a major cause of mortality. Pleurisy occurs in 40% of affected children. Pulmonary complications include lupus pneumonia, pulmonary hemorrhage, interstitial lung disease, and pulmonary hypertension as well as susceptibility to pulmonary infections (Fig. 11) [40, 41]. Lung lesions often progress insidiously and pulmonary fibrosis may exist even in asymptomatic children.

**Fig. 12:**
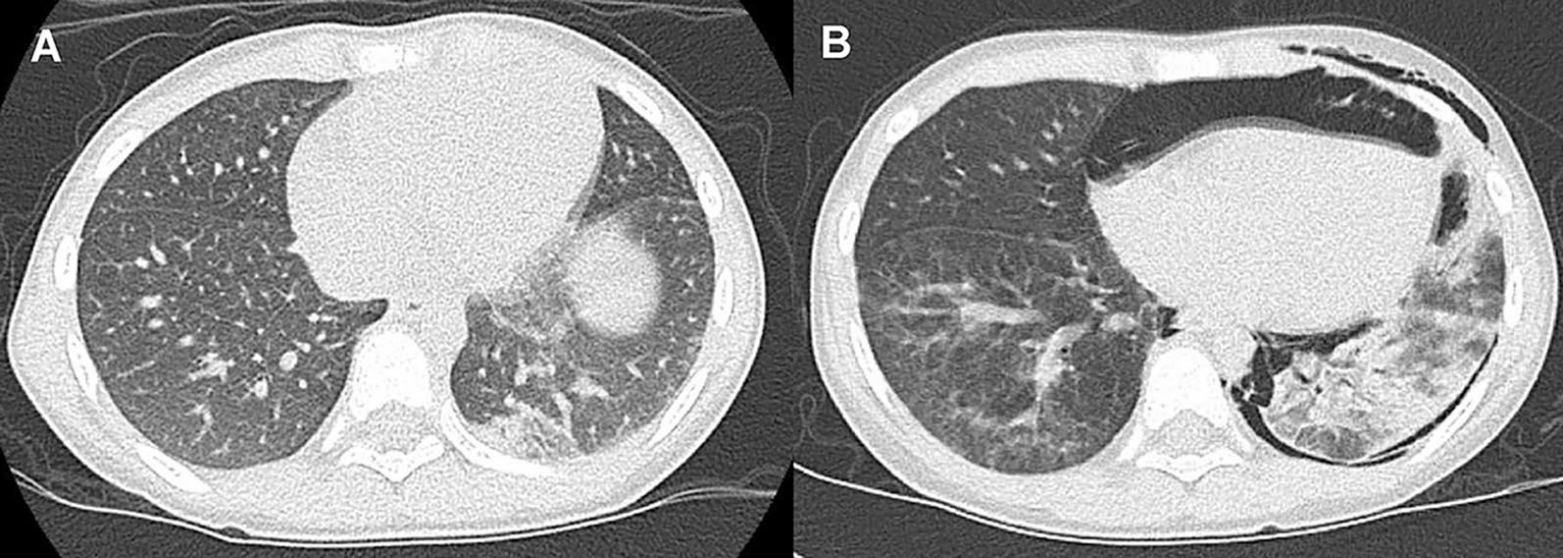
Rapidly progressive interstitial lung disease (RP-ILD) in dermatomyositis. A 5-year-old girl with positive anti-MDA5 antibody. **A** The initial CT showed a pleural based GGO in the left lung base. **B** Six weeks later, she developed respiratory failure. GGOs extended into the wide zones of both lung bases, and complicated by pneumothorax, pneumomediastinum, and subcutaneous emphysema

Pulmonary complications of juvenile dermatomyositis (JDM) include hypoventilation and aspiration pneumonia due to respiratory and swallowing muscle weakness, and intrinsic pulmonary derangement (ILD). JDM is less often complicated by ILD than adult DM; however, it is the major cause of pulmonary morbidities [42]. CT depicts imaging findings of ILD even in asymptomatic children. The early manifestations include infiltrative and reticulogranular shadows or GGOs predominantly seen in the lower and dorsal lungs, while the late consequences include honeycomb lungs with traction bronchiectasis [42]. JDM is complicated by RP-ILD (rapidly progressive interstitial lung disease), which is a lethal complication of DM commonly associated with anti-melanoma differentiation-associated gene 5 (MDA-5) antibody. Individuals with high ferritin and IL18 levels are at risk for RP-ILD, indicating that cytokine aberration plays a crucial role in pathogenesis. Adult patients with RP-ILD are usually amyopathic, while affected children tend to be myopathic. CT findings of RP-ILD are characterized by subpleural curvilinear shadows, peribronchial infiltrates, and GGOs (Fig. 12); however, the early diagnosis of RP-ILD is not easily accomplished. The delayed diagnosis of RP-ILD is fraught with the risk of fatal alveolar damage [43]. Subcutaneous calcinosis and ulcerative lesions due to vascular damage are more common in JDM than in adult DM, while JDM is not at risk for malignant tumors. The timely diagnosis and prompt medical intervention for JDM guarantee a complete cure in about half of affected children.

**Fig. 13:**
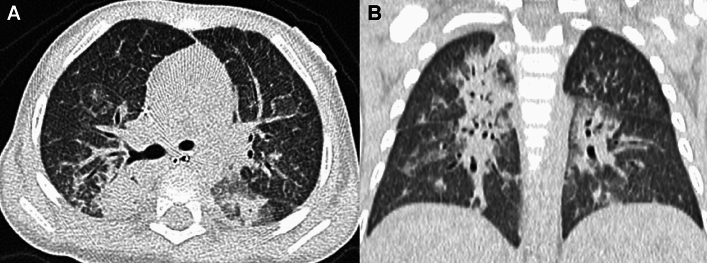
Gaucher disease. An 11-month-old infant. **A**,**B** CT showed diffuse thickening of the interlobar septa and bronchovascular bundles, patchy GGOs, and interstitial infiltrative shadows, giving rise to a crazy paving appearance. Dependent atelectases are seen in both lung bases

Lysosome storage diseases may be associated with pulmonary involvement. The most common disorder is Gaucher disease, which is caused by mutations in the *GBA* gene encoding the lysosomal glucocerebrosidase enzyme. Accumulation of glucocerebrosidase within macrophages occurs primarily in the liver, spleen, and bone marrow, causing hepatosplenomegaly and hypersplenism, and bone involvement with pathological fractures. Gaucher cells (swollen macrophages due to glucocerebrosidase accumulations) can infiltrate the pulmonary alveoli, interstitium, bronchioles, and pulmonary vessels, which may develop pulmonary hypertension. Concurrent liver and lung involvement causes hepatopulmonary syndrome (intrapulmonary shunts), which is the most devastating complication of Gaucher disease [44, 45]. CT shows thickened interlobular septa and GGOs with a crazy paving pattern (Fig. 13). Other findings include reticulo-nodular shadows, thymic enlargement, and swollen mediastinal/hilar lymph nodes. Imaging findings can precede clinical symptoms [44]. Enzyme replacement therapy and substrate reduction therapy may prevent disease progression. Sphingomyelin accumulation due to deficiency of sphingomyelinase in Niemann–Pick disease also creates pulmonary findings similar to those of Gaucher disease.

Langerhans cell histiocytosis (LCH) is a hybrid disease with both neoplastic and inflammatory characteristics with clonal proliferation of bone marrow-derived CD1a, CD207+ dendric cells, and activation of mitrogen-activated protein kinase (MAPK)/extracellular signal-regulated kinases (ERK) signaling. Adult LCH is principally a lung-restricted disease associated with smoking. In contrast, pediatric LCH is a multi-system disorder, in that pulmonary involvement reflects only a part of the disease process.

**Fig. 14:**
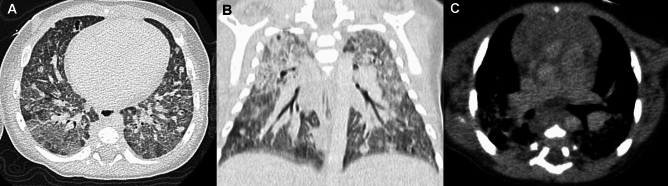
Langerhans cell histiocytosis (LCH). A 2-year-old boy with fever and lymphadenopathy. **A**,**B** CT depicted reticulo-nodular shadows predominantly in the upper and middle lung fields, intermingled with small cavitary lesions. **C** CT also showed thymic enlargement with heterogeneous internal densities and spotty calcifications. The thymic calcifications indicate a high likelihood of LCH

The liver, spleen, and bone marrow are regarded as “risk organs” for high mortality of LCH. The lung is not advocated as a risk organ, but pulmonary lesions commonly occurs in affected children with “risk organ” [46]. Moreover, a subset of affected children may develop rapidly progressive airspace dilatation, leading to respiratory failure and early demise within a year after disease onset [47]. The imaging findings are essentially the same as those of adult LCH, and the upper and middle lung predominance is the rule. The early manifestations are centrilobular nodules and reticular shadows that progress into extensive cyst formation and fibrosis (Fig. 14). The lung cysts vary in size and wall thickness, and usually appears in a round or oval shape but sometimes in an irregular branching pattern. The characteristic “cystic changes” may be obscured by concurrent DIP-like GGOs. In such cases, minimum intensity projection (MinIP) images can highlight the cystic lesions [48]. Rupture of the cyst result in pneumothorax [48]. In addition, thymic involvement eventually causes thymic enlargement and intrinsic calcifications, which may help make the diagnosis. Recently, MEK inhibitors and BRAF inhibitors have been used for LCH, but their efficacy remains controversial [49, 50].

### Disorders of the immunocompromised host

The group includes a large number of congenital and acquired disorders. The comprehensive discussion of this subject is beyond the scope of this review. We discuss here a limited number of well-known disorders.

**Fig. 15:**
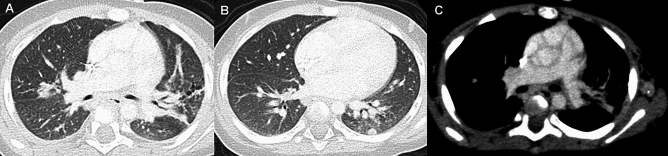
Chronic granulomatous disease. An 11-month-old boy. **A**,**B** CT showed various inflammatory changes, including infiltrative shadows, nodules, masses, and GGOs along with local emphysematous changes in both lungs. **C** Mediastinal, hilar, and axillary lymphadenopathy was evident as well

Chronic granulomatous disease (CGD) is related to genetic defects of NADPH oxidase that prevents synthesis of reactive oxygen species (ROS). Lack of ROS production causes susceptibility to bacterial and fungal infections. The disorder is also associated with granuloma formation as a result of prolonged excessive inflammatory response. CGD is genetically heterogeneous, and inherited either as an X-linked or autosomal recessive trait with a male to female ratio of 3:1. Recurrent infection brings affected children to medical attention at less than 2 years of age. The lung is most commonly affected and often accompanied by infections in the skin, gastrointestinal tract, liver, and bones. Staphylococcus aureus, Aspergillus, Nocardia, fungi, TB and BCG, and Salmonella are common causative agents. Aspergillosis is particularly common in the lung [52]. The imaging findings of acute infection in GGD are diverse, and include infiltrative shadows, GGOs, granular shadows, branching shadows, and miliary nodules. Prolonged inflammation causes not only granuloma formation but also other chronic inflammatory consequences, such as bronchiectasis, thickening of the interlobular septal, cystic changes, fibrosis, honeycomb lungs, lymph nodes swelling, and pleural thickening (Fig. 15). Lung abscesses and pyothorax are also frequent.

**Fig. 16:**
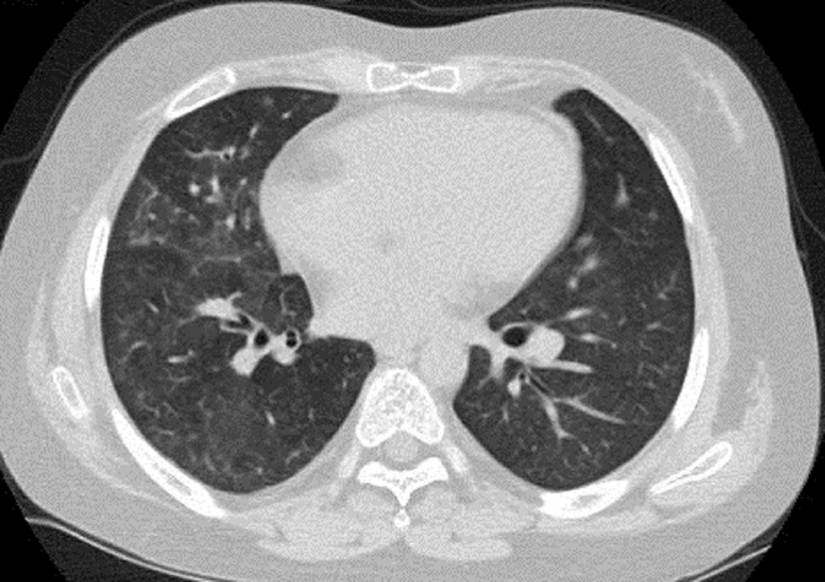
Post-transplant obstructive bronchiolitis. A 15-year-old girl with thalassemia who developed exertional dyspnea 18 months after hematopoietic stem cell transplantation. CT showed a mosaic perfusion pattern or admixture of hyperinflation and patchy GGOs of both lungs, associated with mild bronchial wall thickening

Post-transplant lung diseases occur as a result of immune dysregulation, regardless of solid organ or hematopoietic cell transplantation. Post-transplant lung injury depends on lapse time after intervention, and ranges from capillary leak syndrome and diffuse alveolar hemorrhage in the early phase, to organizing pneumonia in the subacute phase, to bronchiolitis obliterans (BO) in the chronic phase. BO is the most ominous complication. The histological findings of BO are bronchiolitis with lymphocytic infiltration leading to subepithelial fibrosis of the airways and constrictive bronchial lumen narrowing, accompanied by fibrosis and narrowing of small vessels. Hyperinflation, air trapping, and mosaic attenuation on CT help make the early diagnosis of BO (Fig. 16). In particular, biphasic HRCT of inspiration and expiration phases is especially useful to evaluate subtle air trapping.

### Disorders masquerading as interstitial lung disease

Vascular and lymphatic anomalies are addressed as ILDS in the extended classification of chILDs. However, their pathogenesis and treatment totally differ from those of other ILDs.

**Fig. 17:**
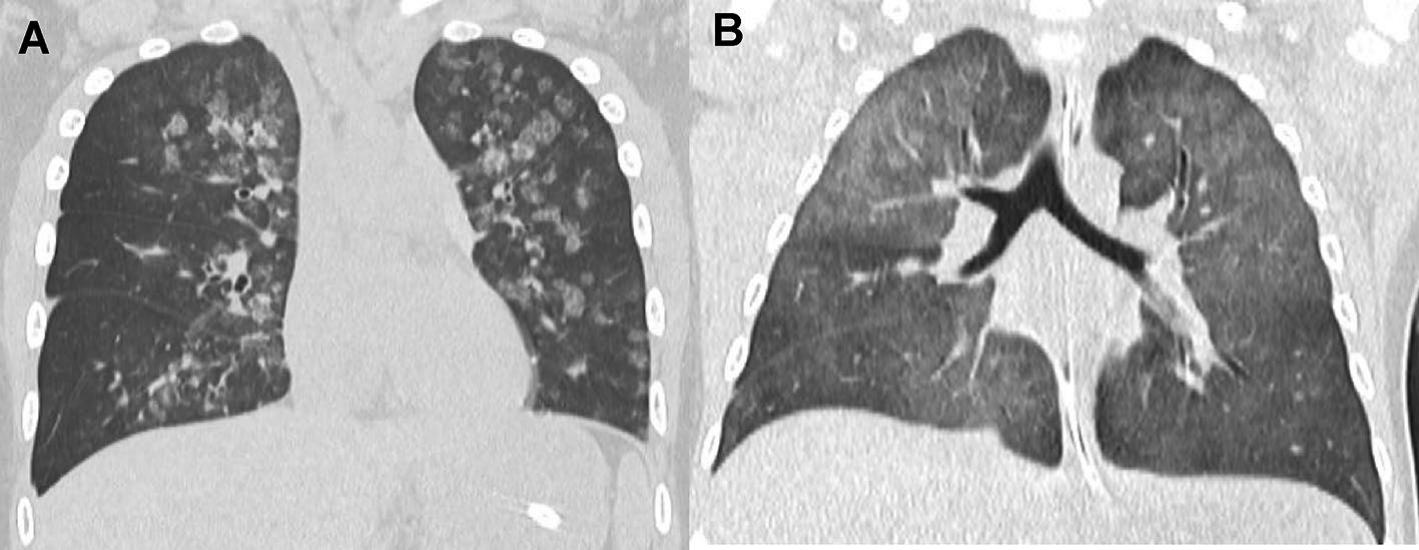
Negative-pressure pulmonary edema (2 cases). **A** An 8-year-old boy after choking. CT sowed ill-defined centrilobular nodules and interlobular septal thickening mainly in the parahilar region. **B** A 9-month-old infant after drowning. CT showed upper lung-dominant diffuse GGOs with subpleural sparing

Pulmonary vascular abnormalities include arterial hypertensive vasculopathy (e.g., idiopathic arteriopathy and vasculopathy secondary to pulmonary parenchymal abnormalities, liver dysfunction, and cardiac disorders with left to right shunt), and congestive vasculopathy (e.g., PVOD, pulmonary vein compression due to mediastinal tumor, left heart failure, and pulmonary vein stenosis or obstruction). The “interstitial” manifestation of vascular abnormalities is related to the thickening of the perivascular interstitium. Another example is interstitial pulmonary edema (e.g., cardiac or noncardiac edema and fluid overload). A relatively child-specific form of pulmonary edema is negative-pressure pulmonary edema associated with aspiration of a foreign body (Fig. 17). The other example is vascular and lymphatic anomalies that we discuss later in this article.

**Fig. 18:**
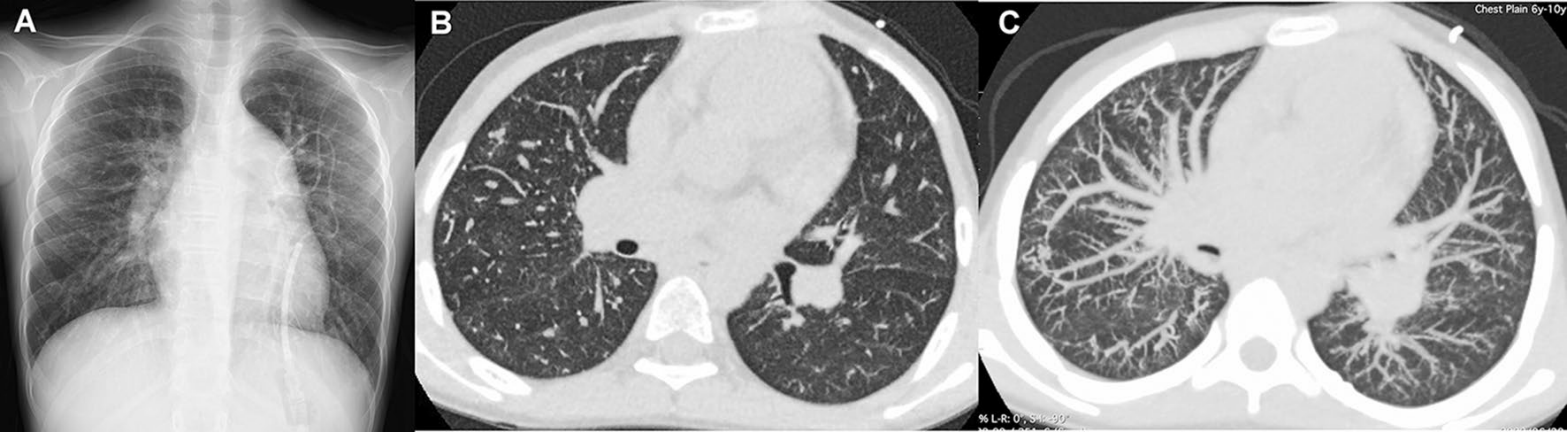
Hereditary hemorrhagic telangiectasia. An 8-year-old girl with decreased exercise tolerance. **A** CXR showed prominence of central and peripheral pulmonary vascular shadows. The PA segment was protruded. **B** CT revealed a number of nodular and tortuous shadows connected to the adjoining vasculatures, suggesting diffuse type AVMs. **C** MIP imaging provided better visualization of continuity of arteries, shunt vessels, and veins

Hereditary hemorrhagic telangiectasia (HHT) or Rendu–Osler–Weber disease is an autosomal dominant disorder with incomplete penetrance and striking intrafamilial phenotypic variabilities. HHT is genetically heterogeneous, and *ACVRL1*, *ENG*, *SMAD4*, and *GDF2* have been identified as causative genes. The major vascular anomaly in HHT is AVM that occurs in the brain, liver, and gastrointestinal tract, as well as in the lung. “Teleangiectasia” is seen in the nasal mucosa and skin. Overall, pulmonary AVM occurs in about half of affected individuals. Lung involvement is more frequent and likely to include multiple AVMs with the in *ENG* mutation. Pulmonary AVMs are often complicated by hypoxemia and sometimes by a sudden onset of paradoxical embolus. CT is employed as pre-embolization imaging for detection of AVMs themselves and identification of all feeding vessels. In the interventional context, pulmonary AVM is divided into a simple type with only one feeding artery, a complex type with two or more feeding arteries, a diffuse type with involvement of all regional arteries, and a telangiectatic type showing a distinctive imaging findings (halo around the AVM) [53]. The feeding arteries and draining veins are differentiated by their morphology and diameter. The arteries tend to be serpentine and small in size, while the veins tend to be relatively straight and larger in size. Maximum intensity projection (MIP) and volume rendering images are useful for detecting small AVMs (Fig. 18).

**Fig. 19:**
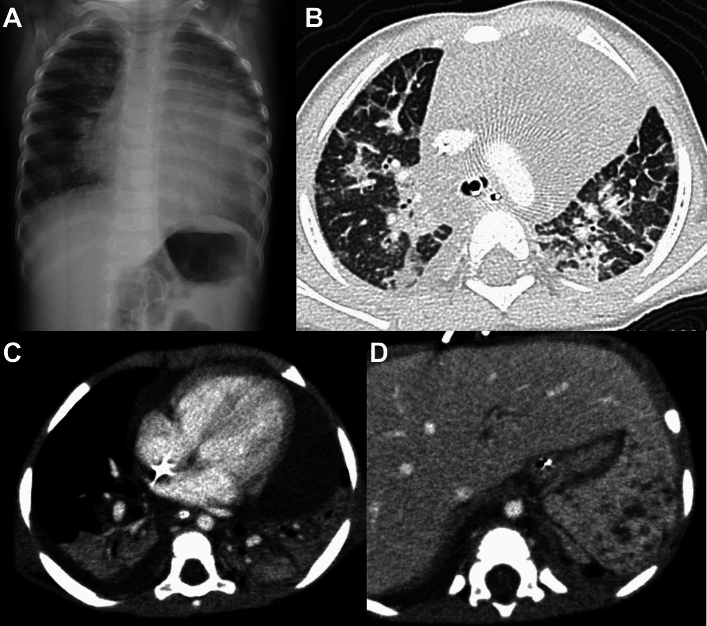
Generalized lymphatic anomaly. A 1-year-old girl with persistent respiratory symptoms. **A** CXR showed enlargement of the cardiac outline and prominent pulmonary interstitial opacities. **B**,**C** CT showed interlobular thickening associated with dependent atelectasis, a small amount of pleural effusion, and a moderate amount of pericardial effusion CT showed interlobular thickening associated with dependent atelectasis, a small amount of pleural effusion, and a moderate amount of pericardial effusion. **D** Abdominal CT revealed splenic lymphangiomatosis

Congenital lymphatic malformations involving the lungs are divided into two categories, (1) congenital lymphangiomatosis (generalized lymphatic anomaly and Gorham-Stout disease) and (2) congenital lymphangiectasia (congenital dilation of lymphatic vessels). Since pulmonary lymphatic vessels are predominant in the vicinity of the pulmonary vessels, interlobular septa, and subpleural interstitum, lymphatic hyperplasia or dilatation masquerade as chILD on radiological grounds. Generalized lymphatic anomaly is the most common type of congenital lymphangiomatosis, producing lymphatic hyperplasia in all organs except the central nervous system. The symptoms and prognosis depend on afflicted organs. In general, when the lung is affected, respiratory failure begins in neonates and infants, and the prognosis is poor. Congenital lymphangiectasia is presumed to be caused by failure of apoptosis of embryonic lymphatic channels, and commonly associated with Turner and Noonan syndromes, as well as with left-sided heart obstructive diseases (e.g., total anomalous pulmonary venous connection and hypoplastic left heart syndrome) [54]. Even if affected fetuses survive, they have postnatal respiratory morbidities, including massive chylothorax due to fragile embryonic lymphatic channels and lethal hypoxemia due to alveolar effacement from dilated lymph vessels. Lymphangiomatosis and lymphangiectasia manifest in bilateral diffuse increased densities on plain radiographs. CT shows diffuse thickening of bronchovascular bundles and interlobular septa, commonly associated with pleural effusion, hyperplasia and increased densities of the mediastinal soft tissue (Fig. 19). Heavily T2-weighted MR imaging can capture the entire picture of lymphatic malformation.

## Conclusion

Childhood ILDs (chILDs) of infancy onset are exceedingly rare and the imaging findings are poorly understood. However, awareness of the “unexplained” ILDs should increase the likelihood of the accurate diagnosis for affected infants, which enables to more precisely understand the pathogenesis and develop efficient medical intervention of the unsolved disorders. The disease spectrum of ILD of childhood onset overlaps with that of adult ILD. However, it is noteworthy that even the same disease presents differently in affected children and adults.
